# Optimized Back Propagation Neural Network Using Quasi-Oppositional Learning-Based African Vulture Optimization Algorithm for Data Fusion in Wireless Sensor Networks

**DOI:** 10.3390/s23146261

**Published:** 2023-07-09

**Authors:** Alaa A. Qaffas

**Affiliations:** Department of MIS, College of Business, University of Jeddah, Jeddah 21589, Saudi Arabia; aaqaffas@uj.edu.sa

**Keywords:** African Vulture Optimization Algorithm, back propagation neural network, cluster-based routing, data fusion, quasi-oppositional learning, wireless sensor networks

## Abstract

A Wireless Sensor Network (WSN) is a group of autonomous sensors geographically distributed for environmental monitoring and tracking purposes. Since the sensors in the WSN have limited battery capacity, the energy efficiency is considered a challenging task because of redundant data transmission and inappropriate routing paths. In this research, a Quasi-Oppositional Learning (QOL)-based African Vulture Optimization Algorithm (AVOA), referred to as QAVOA, is proposed for an effective data fusion and cluster-based routing in a WSN. The QAVOA-based Back Propagation Neural Network (BPNN) is developed to optimize the weights and threshold coefficients for removing the redundant information and decreasing the amount of transmitted data over the network. Moreover, the QAVOA-based optimal Cluster Head Node (CHN) selection and route discovery are carried out for performing reliable data transmission. An elimination of redundant data during data fusion and optimum shortest path discovery using the proposed QAVOA-BPNN is used to minimize the energy usage of the nodes, which helps to increase the life expectancy. The QAVOA-BPNN is analyzed by using the energy consumption, life expectancy, throughput, End to End Delay (EED), Packet Delivery Ratio (PDR) and Packet Loss Ratio (PLR). The existing approaches such as Cross-Layer-based Harris-Hawks-Optimization (CL-HHO) and Improved Sparrow Search using Differential Evolution (ISSDE) are used to evaluate the QAVOA-BPNN method. The life expectancy of QAVOA-BPNN for 500 nodes is 4820 rounds, which is high when compared to the CL-HHO and ISSDE.

## 1. Introduction

The fast development of electronic component manufacturing and wireless communication encourages WSN application using wireless sensor nodes, because of the inherent features such as strong adaptability, versatility and low-cost [1]. Sensors of WSNs observe the different environmental hazards created in unattended/remote areas. These sensors analyze the various environmental factors such as the temperature, humidity, pressure and so on for identifying various catastrophic conditions before their incidence [2]. The sensors of WSN have the responsibility to collect and maintain the potential data for achieving the mandatory decision-making process that is required to be effectively performed by BS [3]. This sensor node transmits the observed information as data packets before transmitting to the central base station [4]. WSN plays an essential role in various fields such as smart home, medical and health, military reconnaissance, safety and stability maintenance, surveying and mapping, agricultural irrigation, industrial monitoring and smart transportation [5].

Each node in the WSN is installed with limited storage, energy and processing capacities. Therefore, energy preservation is considered as an important task for improving the life expectancy, and clustering is considered as the most energy-saving approach in the WSN [6,7,8]. A clustering approach is used to group the sensors, namely clusters where each cluster has one header node, i.e., Cluster Head (CH). CH collected the data from the member nodes of the cluster and transmits them to the BS [9,10]. The direct transmission of data from all sensors is not efficient while broadcasting the data packets. The adjacent sensors have highly correlated data gathered in the network, which has greatly redundant raw data. The transmission of all raw data to the BS causes network congestion and high-power consumption, which create a huge impact on life expectancy. Therefore, the data fusion approach is considered in the application of the WSN [11,12]. The main principle of data fusion is to combine the data received from various sensors, and the fused information is forwarded to BS [13,14,15]. An efficient data fusion is depending on the sensors delivery information where reliability and efficiency are the key parameters [16].

The contributions are as follows:

1. The QAVOA-based CHN and route discovery are carried out for performing reliable data transmission over the network. The conventional African Vulture Optimization Algorithm (AVOA) is converted into QAVOA by incorporating QOL, which helps to enhance the population diversity for achieving the optimum solutions.
2. Moreover, a QAVOA-optimized BPNN is used to perform an effective data fusion for eliminating the redundant information and minimizing the amount of information, which helps to minimize node’s energy usage and enhance network’s life expectancy.

The remaining paper is sorted as follows: Section 2 provides the related works about the data fusion and cluster-based routing over the WSN. The detailed information about the QAVOA-BPNN is provided in Section 3 while the outcomes of the QAVOA-BPNN are given in Section 4. Further, the conclusion is provided in Section 5.

## 2. Related Work

The related works about the data fusion and cluster-based routing over the WSN are provided in this section.

### 2.1. Data Fusion in WSN

In general, the data fusion [17,18] is accomplished by each node discarding multiple copies of similar data. Therefore, the data fusion is used to remove the data redundancies of the network.

Cao et al. [19] presented the Extreme Learning Machine (ELM) with a bat algorithm for performing the data fusion over the WSN, whereas the sensors were defined as neurons of ELM. The observed data were gathered by using the sensors of the mobile heterogeneous WSN, and the gathered data were combined with the clustering route. The capacity of ELM was enhanced by optimizing its weights and thresholds using a bat algorithm, which was used to enhance the generalization capacity. The developed ELM with bat algorithm was considered only the distance and energy parameters.

Liu et al. [20] developed the Hybrid Delay-aware Clustering (HDC) for ensuring the intelligent data fusion approach in WSN. The advantages of cluster architecture from single and multiple layers were found in the HDC, and cluster patterns were adaptively chosen for obtaining the balance among the energy usage and delay. The energy usage of WSN was balanced by using the intelligent energy efficient clustering and dynamic cluster head reselection approach. The distance and residual energy parameters were only considered during the clustering and recombination stages. The life expectancy of the network was high when it was processed with HDC.

Gavel et al. [21] used the benefits of Grey Model (GM) and Kernel-based ELM (KELM) for developing the integration of data aggregation-based data fusion and an effective fault identification over the WSN. The single datum pattern was recorded by eliminating the repetitive information collected from the sensors by using the GM. Further, higher confidentiality of the network was maintained by accomplishing the fault identification using a trained KELM. The longevity and reliability of the WSN was enhanced by using the developed GM-KELM. The developed GM-KELM was mainly concentrated on data fusion, however an effective routing over the network was required to be considered for a reliable data broadcasting over the network.

Long et al. [22] developed the belief structure-based data fusion for reducing the attributes of the multi-granulation rough set. Next, the transitivity and consistency were examined by developing the architecture of the positive region, evidence and belief. Further, the reduction carried out in this work was used to enhance the efficiency of computations. The attribute discretization in the belief structure created a huge impact on the overall efficiency.

Luo [23] presented the adaptive weighted D–S evidence theory and mass deep auto-encoder for performing the data fusion over the WSN. The data features of each cluster were extracted and classified by using feature extraction and classification. Next, fused features of the same class were broadcasted to the sink node. The performance of the autoencoder was improved, only when increasing the number of layers during the data fusion.

### 2.2. Cluster Based Routing

This section provides the information about the different research, which was carried out based on the swarm-intelligence-based approaches.

There are different optimizations such as oppositional artificial fish swarm-based clustering with improved moth flame optimization-based routing [24], Particle Swarm Optimization (PSO) [25], shuffled frog leaping algorithm [26], opposition-based learning with gray wolf optimization-based clustering [27], Re-position PSO (RPSO) [28], Cross-Layer-based Harris Hawks Optimization (CL-HHO) [29] and Improved Sparrow Search using Differential Evolution (ISSDE) [30]. Some of the aforementioned approaches are explained as follows: Elshrkawey et al. [28] developed the RPSO for developing efficient routing in the WSN. The RPSO was used to alter the conventional expressions of location, velocity and inertia weight. This developed RPSO was used to select optimal CHs for increasing the lifetime. Xue et al. [29] presented the clustering using k-medoids with improved artificial-bee-colony and routing using CL-HHO for enhancing the quality-of-service parameters. A cross-layer-based optimal routing issue was developed for overcoming the issue related to the power asymmetry. The delay and power usage were minimized by using the cross-layer routing approach. Kathiroli and Selvadurai [30] developed the ISSDE for resolving the issue related to the CH selection. The inter-cluster communication was effectively achieved by using the searching and antipredator nature of sparrows. The sparrow search was used to discover the shortest path among CH and BS. However, these optimization-based cluster-based routing approaches were not considered as effective data fusion approaches.

A distributed Internet of Things (IoT) system for monitoring CO_2_ emissions has been designed and implemented in [31]. The LoRaWAN communication protocol used long-range radio frequency technology. In [32], a concept for an IoT wide-area communication system is presented, which was installed within the operator’s licensed macrocellular band and appropriate for low-energy, complexity, and traffic IoT modules. In [33,34], a testing on CoAP Multi Factor Authentication Mechanism with Reputation for IoT Constrained Devices and a novel fair scalable relay control scheme for IoT have been employed.

The data-driven surrogate model with latent data assimilation has been shown by Sibo Cheng et al. [35]. By combining machine learning, model reduction, and data assimilation approaches, this work intends to enable real-time integration of the observation data for improving predictions supplied by the data-driven surrogate model in order to improve the effectiveness and accuracy of the fire monitoring system. The main goal was to accurately foresee how burned regions would change in order to indicate the emergence of wildfires. Therefore, it was crucial to perform Data Assimilation (DA) and machine learning prediction at a reasonable cost. The suggested approach’s effectiveness is ensured by reduced-order modelling and a machine learning surrogate model, and the system may change the simulation with observations thanks to data assimilation. Reduced-order surrogate models and a DA approach that incorporates real-time data from various physical regions make up the system Sibo Cheng et al. [36] described. Then, we process the variational DA with a minimal computational cost, employ local smooth surrogate functions that connect the space of encoded system variables and the one of current observations. The new technique, known as generalized latent assimilation, benefits from both the precision of data assimilation and the efficiency offered by reduced order modelling. The novel method has been tested on a two-phase liquid flow application in high dimensions with non-linear observation operators that cannot be handled by existing latent assimilation techniques.

## 3. QAVOA-BPNN Method

**Figure 1:**
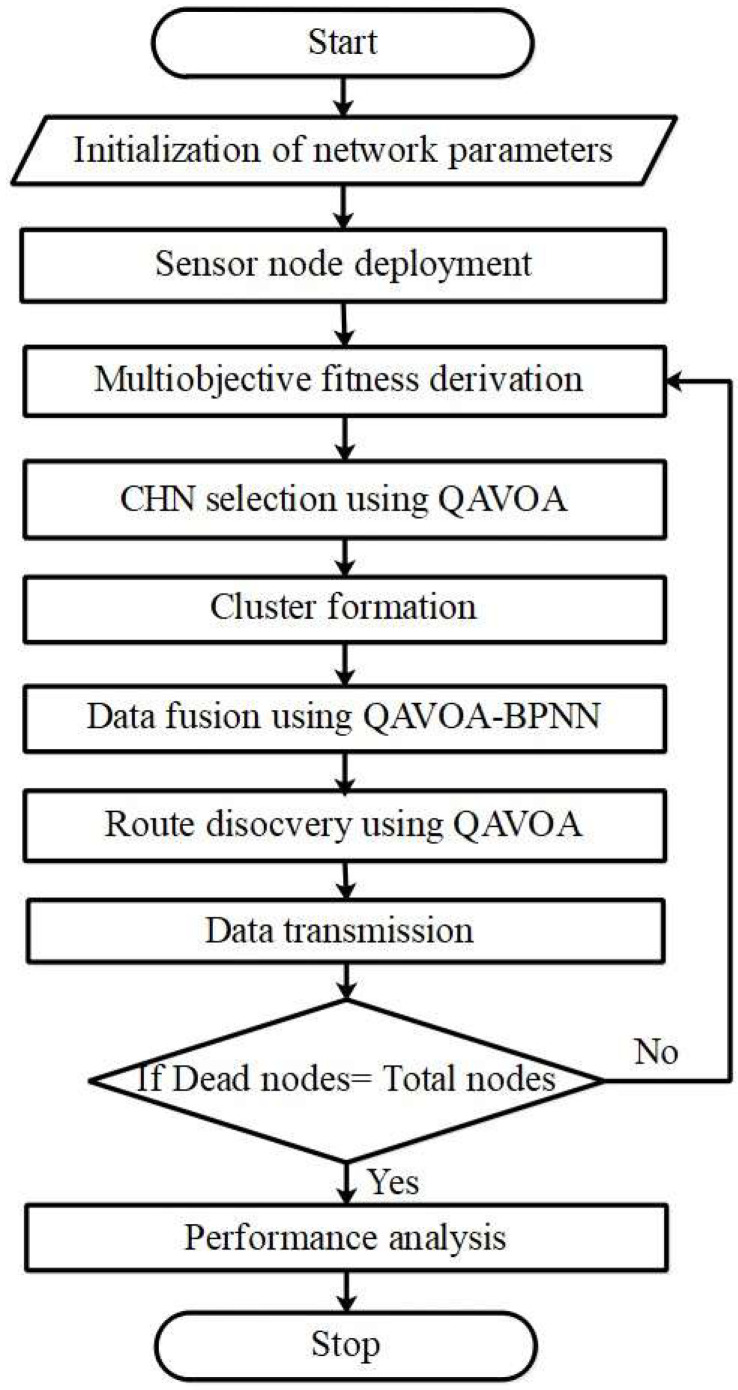
Flowchart for QAVOA-BPNN method.

This research performs an effective data fusion and cluster-based routing for improving the WSN performances. The BPNN is used for performing the data fusion where the weights and threshold coefficients are optimized by QAVOA. Moreover, the cluster-based routing using QAVOA is helps to choose optimum CHN and path for performing the reliable transmission. The data fusion performed by QAVOA-BPNN helps to minimize the amount of broadcasted data and eliminate the redundant data, which returns the lesser energy usage. Therefore, the lesser energy usage is used to enhance the network’s life expectancy. The flowchart for the QAVOA-BPNN is shown in Figure 1.

### 3.1. Sensor Initialization

The sensors are randomly positioned in the network followed by multi-objective QAVOA, which is used for searching the CHs. The clusters are created once the CHs are chosen in the network. Moreover, the QAVOA-based route discovery is accomplished for performing the data broadcasting in WSN. The detail about the CHN and route discovery are provided in the following sections.

### 3.2. QAVOA Based CHN Discovery

The optimum CHs from the normal nodes are chosen using the QAVOA. The conventional AVOA [37] is motivated by the hunting action of African vultures, whereas the strategy of QOL is incorporated for maximizing the population diversity, which leads to achieving the better searching capability.

#### 3.2.1. Initialization of CHN Discovery

The initial solutions, i.e., vultures, are fixed with a set of nominee sensors to be selected as CH. The vultures are set with a random sensor between 1 and M, where the total sensors in the WSN are M. Consider, the i th vulture of QAVOA is $P_{i}=\left(P_{i,1},\;P_{i,2},\ldots ,\;P_{i,d}\right)$, where QAVOA’s dimension is denoted as d, which is equal to the number of CHs.

#### 3.2.2. Iterative Process

The inputs from the initialization are given to an iterative process for identifying the optimum CHs.

##### Quasi-Oppositional Learning for Enhancement

In general, the opposition learning is used to create the possible solutions, examine the relative solutions and choose the optimum candidate solutions for improving the capacity of searching to solve the optimization issue. Here, the QOL is developed according to the opposite number as expressed in Equation (1).

(1) $$P=rand\left(\frac{lb+ub}{2},\;P^{o}\right)$$

where, the middle point of the lb and ub interval is denoted as (lb+ub)/2 and the random number among (lb+ub)/2 and $P^{o}$ is denoted as $rand\left(\left(lb+ub\right)/2,\;P^{o}\right)$. Equation (2) denotes the $P^{o}$, i.e., opposition learning.

(2) $$P^{o}=lb+ub-P$$

where, the point in n dimensional space is denoted as P.

##### Classification of Population

The population is separated in to three classes where the fitness value is utilized for defining whether the vulture location is good or not. Therefore, the 1st group is defined as the first optimal solution for all vultures, the 2nd group is the 2nd optimal solution, and the remaining is defined as the third group. The fitness for each population is computed, once the initialization is performed for African vultures. The formula for vulture grouping is shown in Equation (3).

(3) $$R_{i}^{t}=\{\begin{array}{c} Top\;vulture_{1}^{t},\;\;\;\;\;if\;P_{i}^{t}=L_{1}\\ Top\;vulture_{2}^{t},\;\;\;\;\;if\;P_{i}^{t}=L_{2} \end{array}$$

where, the $Top\;vulture_{1}$ and $Top\;vulture_{2}$ denote the optimal and suboptimal solutions for the population; random numbers generated within the range of [0, 1] are denoted as $L_{1}$ and $L_{2}$, whereas the sum of two numbers are 1. Moreover, $P_{i}$ is achieved from the roulette wheel strategy as shown in Equation (4).

(4) $$P_{i}^{t}=\frac{f_{i}^{t}}{\sum_{i=1}^{m}f_{i}^{t}}$$

where, the vulture’s fitness is denoted as $f_{i}$ and the total amounts of vultures in the 1st and 2nd groups are denoted as m.

##### Vulture Starvation Rate

An African vulture’s strength is denoted by the starvation rate of QAVOA. The vulture flies too far searching for the food when it is not hungry. On the other hand, the vulture does not have the strength to fly far when it is hungry. Consequently, the hungry vultures certainly become violent and surround strong vultures to obtain food instead of discovering food themselves. The exploration and development stage of vultures are varied based on the starvation rate that is expressed in Equations (5) and (6).

(5) $$t=h\times \left({sin}^{w}\left(\frac{\pi }{2}\times \frac{iteration_{i}}{maxiterations}\right)+cos\left(\frac{\pi }{2}\times \frac{iteration_{i}}{maxiterations}\right)-1\right)$$

(6) $$F=\left(2\times rand_{1}+1\right)\times z\times \left(1-\frac{iteration_{i}}{maxiterations}\right)+t$$

where, the starvation rate of vultures is denoted as F; iteration denotes the current iteration; the total amount of iterations is denoted as maxiterations; and the random value between [−1, 1] is denoted as z. The vulture is hungry when z is less than 0; the vulture is full when z is greater than 0. The random value between [−2, 2] is denoted as h and the random value between [0, 1] is denoted as $rand_{1}$. The probability a vulture enters to explore is affected by the preset value w; the probability of entering to explore is high when the w is high and vice versa. The vulture observes the various areas for locating food when the |F|≥1; otherwise, the vulture searches in adjacent locations, when |F|<1. In this stage, the QAVOA is processed under the development stage.

##### Exploration Stage

Generally, the African vulture consumes some time for determining the position of remaining populations and then flies a higher distance for searching for food. Two different exploration approaches are designed and the strategy is defined by using $P_{1}$. This parameter has a fixed value in the range of [0, 1] before the search function. The exploration approach is defined by comparing the $rand_{P1}$ generated in the range of [0, 1] with $P_{1}$. Equation (7) shows the exploration of vultures.

(7) $$P_{i}^{t+1}=\{\begin{array}{l} R_{i}^{t}-D_{i}^{t}\times F\;\;\;\;\;\;\;\;\;\;\;\;\;\;\;\;\;\;\;\;\;\;\;\;\;\;\;\;\;\;\;\;\;\;\;\;\;\;\;\;\;\;\;\;\;\;\;\;\;\;\;\;\;\;if\;P_{1}\geq rand_{p1}\\ R_{i}^{t}-F+rand_{2}\times \left(\left(ub-lb\right)\times rand_{3}+lb\right)\;\;\;\;\;if\;P_{1}<rand_{p1} \end{array}$$

where, $rand_{2}$ and $rand_{3}$ are the value generated among [0, 1]; the upper and lower boundaries are denoted as ub and lb, respectively, and the distance among the current optimal vulture and vulture is denoted as $D_{i}^{t}$, which is expressed in Equation (8).

(8) $$D_{i}^{t}=\left|X\times R_{i}^{t}-P_{i}^{t}\right|$$

where, X is the distance vector, i.e., the vulture which randomly moves to protect the food from other vultures, computed by 2×rand.

##### Development Stage (Initial Stage)

The vultures are entered into the initial development stage for eliminating the imbalance among the exploration and development capacities, when the |F| is among 0.5 and 1, for the determination of vultures that either participate for food or spiral. If the vulture is processed in the initial development stage, the random value $randt_{P2}$ is created between [0, 1] before the vulture moves to another place. The vulture spirals to the remaining positions, when the $randt_{P2}$ is less than $P_{2}$; otherwise, it competes for food.

##### Compete for Food

The vulture starts to think fully when |F| is among 0.5 and 1. If the vultures are gathered in this condition, the strong vultures are not ready to share the food, whereas the weak vultures try to reduce the strong vulture’s energy. Subsequently, the weak vultures gather around the strong ones and provoke small battles for obtaining the food. According to the aforementioned action, the vulture location update is shown in Equation (9).

(9) $$P_{i}^{t+1}=D_{i}^{t}\times \left(F+rand_{4}\right)-\left(R_{i}^{t}-P_{i}^{t}\right)$$

where, the random number between [0, 1] is $rand_{4}$

##### Spiral Flying

The strong vulture and those expressing competition for food fly in a spiral in the air, when 0.5<|F|<1 and $rand_{P2}\geq P_{2}$. The spiral expressed is created along with the aforementioned 2 groups of vultures. Equations (10) and (11) performed the spiral flying. Equation (12) shows the vulture’s location update.

(10) $$S_{1}^{t}=R_{i}^{t}\times \left(\frac{rand_{5}\times P_{i}^{t}}{2\pi }\right)\times cos\left(P_{i}^{t}\right)$$

(11) $$S_{2}^{t}=R_{i}^{t}\times \left(\frac{rand_{6}\times P_{i}^{t}}{2\pi }\right)\times sin\left(P_{i}^{t}\right)$$

(12) $$P_{i}^{t+1}=R_{i}^{t}-\left(S_{1}^{t}+S_{2}^{t}\right)$$

where, the random numbers between [0, 1] are $rand_{5}$ and $rand_{6}$.

##### Development Stage (Later Stage)

All vultures are full when the starvation rate is less than 0.5, but the optimal 2 types of vultures become hungry and weak after flying for long distances. The vulture attacks for the food and various vultures collect the same food. Next, the $P_{3}$ between [0, 1] is utilized for determining whether the vulture shows aggregation or aggressive action. If the vulture is processed under the development process, a $rand_{P3}$ generated among [0, 1] is created before vulture migration. The vulture is processed under aggressive action, when the $rand_{P3}$ is equal or greater than $P_{3}$; otherwise, the vulture is processed under aggregation.

##### Food Source Aggregation

The vultures participate for food to avoid starvation and many flock to the same food source. Equations (13) and (14) show the vulture’s motion while the location update is performed by using Equation (15).

(13) $$A_{1}^{t}=Top\;vulture_{1}^{t}-\frac{Top\;vulture_{1}^{t}\times P_{i}^{t}}{Top\;vulture_{1}^{t}\times {\left(P_{i}^{t}\right)}^{2}}\times F$$

(14) $$A_{2}^{t}=Top\;vulture_{2}^{t}-\frac{Top\;vulture_{2}^{t}\times P_{i}^{t}}{Top\;vulture_{2}^{t}\times {\left(P_{i}^{t}\right)}^{2}}\times F$$

(15) $$P_{i}^{t+1}=\frac{A_{1}^{t}+A_{2}^{t}}{2}$$

##### Fierce Competition for Food

**Figure 2:**
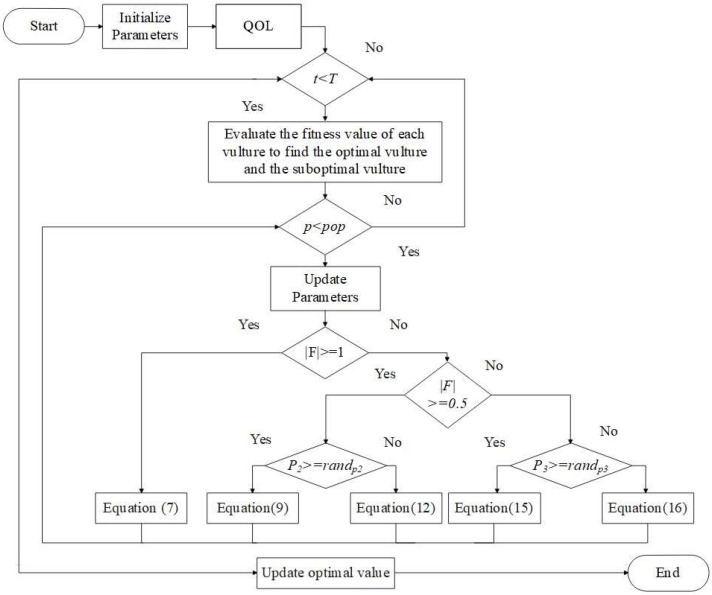
Flowchart for QAVOA.

Simultaneously, the remaining vultures are violent and fly to the leader vulture at various points to obtain the food using Equation (16).

(16) $$P_{i}^{t+1}=R_{i}^{t}-\left|R_{i}^{t}-P_{i}^{t}\right|\times F\times Levy\left(d\right)$$

where, the function’s dimension is denoted as d and the Levy flight mechanism is denoted as Levy(.). The flowchart for QAVOA is shown in Figure 2.

#### 3.2.3. Fitness of QAVOA for CH Discovery

A QAVOA considers the various fitness metrics such as residual energy $\left(fm_{1}\right)$, neighbor node distance $\left(fm_{2}\right)$, sink distance $\left(fm_{3}\right)$, CHN balancing factor $\left(fm_{4}\right)$ and node degree $\left(fm_{5}\right)$. The fitness formulated for the QAVOA is expressed in Equation (17).

(17) $$f=\psi_{1}\times fm_{1}+\psi_{2}\times fm_{2}+\psi_{3}\times fm_{3}+\psi_{4}\times fm_{4}+\psi_{5}\times fm_{5}$$

where, the weight values assigned for each fitness metric are represented as $\psi_{1}-\psi_{5}$. These multiple fitness metrics are explained as follows:

- The CHN’s energy consumption is essential due to its various functions such as data collection, fusion and broadcasting over the network. CHNs with high energy are preferred for performing the reliable data transmission. The residual energy is expressed in Equation (18).
  (18) $$fm_{1}=\sum_{i=1}^{d}\frac{1}{E_{CHN_{i}}}$$
  where, $E_{CHN_{i}}$ is the residual energy of ith CHN.
- Equations (19) and (20) show the neighbor node distance and sink distance that are the distance among the sensors and distance between CHN and BS. The energy consumption of the sensors is directly proportional to the distance, therefore the CHN with a lesser distance is chosen in WSN.
  (19) $$fm_{2}=\sum_{j=1}^{d}\left(\sum_{i=1}^{CMN_{j}}dis\left(M_{i},CHN_{j}\right)/CMN_{j}\right)\;$$
  (20) $$fm_{3}=\sum_{i=1}^{d}dis\left(CHN_{i},\;BS\right)\;$$
  where, the $CMN_{j}$ is the member node from the jth cluster; the distance between the ith sensor and the jth CHN is denoted as $dis\left(M_{i},CHN_{j}\right)$ and the distance between the ith CHN and BS is denoted as $dis\left(CHN_{i},\;BS\right)$.
- The network has the possibility of generating huge clusters with small clusters. Equation (21) expresses the CHN balancing factor, which is used to achieve the balance among the clusters for obtaining an effective balancing of energy in WSN.
  (21) $$fm_{4}=\sum_{i=1}^{d}\frac{AN}{d}-CMN_{i}$$
  where, the alive nodes in the network are denoted as AN.
- A number of nodes that belong to the next hop is represented as node degree, which is expressed in Equation (22).
  (22) $$fm_{5}=\sum_{i=1}^{d}CMN_{i}$$

These fitness metrics are utilized for selecting optimum CH between the Cluster Member Nodes (CMNs). The failure node is avoided by considering the energy that is used to avoid packet drop. The CHN with a lesser distance is obtained by considering the distance factor that helps to lessen the energy usage over the network, whereas the node degree is also used to minimize the energy [38]. Further, the energy balancing among the clusters is achieved by using the CHN balancing factor.

### 3.3. Generation of Clusters

The CMNs are assigned to an adequate CHNs, once the selection of CHN is made by using QAVOA. Equation (23) shows the potential function used for generating the clusters.

(23) $$Potential\;function\;\left(M_{i}\right)=\frac{E_{CHN}}{dis\left(M_{i},CHN\right)}$$

After identifying the CHNs, the data fusion is performed by using QAVOA-BPNN for the data received from respective CMNs.

### 3.4. Data Fusion Using QAVOA-BPNN

This section shows the optimized BPNN using QAVOA to accomplish the data fusion, once CHN receives the data from CMNs. The elimination of unwanted data and the minimization of broadcasted data quantities help to minimize the energy and life expectancy. The BPNN [39] architecture is a three-layer neural network comprised of an input layer, hidden layer, and output layer [40]. The input and output signals for QAVOA-BPNN are $U=\left[u_{1},\;u_{2},\ldots ,u_{ni}\right]$ and $Y=\left[y_{1},\;y_{2},\ldots ,y_{no}\right]$, respectively, and nn, nh and no are the number of neurons in the input, hidden and output layers, respectively.

Equations (24) and (25) show the outputs of the hidden and output layers, respectively. An activation function of the hidden layer and output layer $\left(AF_{v}\left(v\right)\right)$ is expressed in Equation (26).

(24) $$h_{j}=AF_{v}\left(\sum_{i=1}^{nn}wn_{ij}u_{i}+b_{j}\right)$$

(25) $$y_{k}=AF_{v}\left(\sum_{j=1}^{nh}wn_{ij}h_{j}+b_{k}\right)$$

(26) $$AF_{v}\left(v\right)=\frac{1}{1+exp\left(-v\right)}$$

where, the output of neuron j for the hidden layer and neuron k for the output layer are denoted as $h_{j}$ and $y_{k}$, respectively; the weight value between the ith neuron of the input layer and the jth neuron of the hidden layer is denoted as $wn_{ij}$; the weight value between the jth neuron of the hidden layer and the  kth neuron of the output layer is denoted as $wn_{jk},\;\mathrm{and}$ the bias values of the jth neuron of the hidden layer and the kth neuron of the output layer are denoted as $b_{j}$ and $b_{k}$. The output is generally varied from the expected outcome; therefore, the error function is developed as shown in Equation (27).

(27) $$e_{err}=\frac{1}{2}\sum_{k=1}^{no}{\left(y_{k}-{y^{\prime }}_{k}\right)}^{2}$$

where, the expected outcome of neuron k in the output layer is denoted as ${y^{\prime }}_{k}$. The error is gradually minimized by adjusting the weight and bias according to backward error propagation. Equations (28)–(31) shows the weights and the bias adjustments.

(28) $$wn_{ij}\left(t+1\right)=wn_{ij}\left(t\right)-\eta \frac{\partial e_{err}}{\partial wn_{ij}\left(t\right)}$$

(29) $$wn_{jk}\left(t+1\right)=wn_{jk}\left(t\right)-\eta \frac{\partial e_{err}}{\partial wn_{jk}\left(t\right)}$$

(30) $$b_{j}\left(t+1\right)=b_{j}\left(t\right)-\eta \frac{\partial e_{err}}{\partial b_{j}\left(t\right)}$$

(31) $$b_{k}\left(t+1\right)=b_{k}\left(t\right)-\eta \frac{\partial e_{err}}{\partial b_{k}\left(t\right)}$$

where, the learning rate is denoted as h, which is required to be set in optimum for enhancing the training speed and the amount of training times is denoted as t.

The QAVOA is adopted in BPNN for optimizing the weights and threshold coefficients for enhancing the learning capacity and local extremum. The $e_{err}$ is considered as a fitness function for QAVOA that is expressed in Equation (27). The iterative process used to find the optimum weight and threshold coefficients is already detailed in Section 3.2.2.

### 3.5. Route Discovery Using QAVOA

The QAVOA-based route discovery is performed by optimizing it with distance, energy and node degree factors. The following steps depict the route discovery using QAVOA.

1. At first, the vultures are initialized with the possible paths from the transmitter CHN to BS, whereas the dimension of each vulture is equal to the number of relay CHs of the route.
2. The location update is accomplished by using the iterative process detailed in previous sections that was utilized to return to the optimum path based on the fitness metrics.
3. The considered multiple fitness metrics are residual energy, sink distance and node degree. The fitness metric for QAVOA-based route discovery is expressed in Equation (32).
  (32) $$f=\varphi_{1}\times \sum_{i=1}^{d}\frac{1}{E_{CHN_{i}}}+\varphi_{2}\times \sum_{i=1}^{d}dis\left(CHN_{i},\;BS\right)+\varphi_{3}\times \sum_{i=1}^{d}CMN_{i}$$
  where, weight values allocated to fitness are $\varphi_{1}$−$\;\varphi_{3}$. The QAVOA-based route discovery is used to perform the optimum route discovery in WSN.

## 4. Results and Discussion

The results and discussion of the QAVOA-BPNN are shown in this section. The design and execution of QAVOA-BPNN are carried out by using the MATLAB R2020b software. Here, the system is configured with 16 GB RAM and i5 processor. The 500 nodes are considered as randomly deployed in the area of 500 m×500 m, whereas the simulation parameters are shown in Table 1. The performance of the proposed QAVOA-BPNN approach varies with different network topologies and traffic patterns, because the traffic was distributed by each and every node present in the network, where the network performance starts to decrease when the traffic rises. Therefore, the network performance varies according to the traffic and topology.

**Figure 3:**
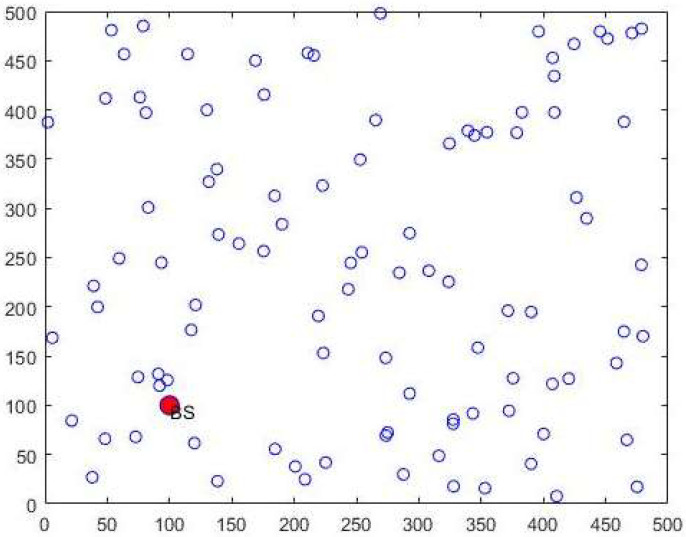
Node distribution is WSN.

The distribution of nodes in WSN is depicted in Figure 3. Where, the red color nodes indicate the base station. In performance analysis, this research has shown the extent to which the proposed QAVOA-BPNN has been capable to deliver the required node density that is developed from the network lifetime analysis. The performance analysis of the network lifetime parameter is specified in the section below. Every node present in the network contains an equivalent priority for transmitting the data. The inequalities of internal nodes are calculated by measuring the range to which the distribution inside an economy diverges from a perfect equal distribution.

### 4.1. Performance Analysis

**Figure 4:**
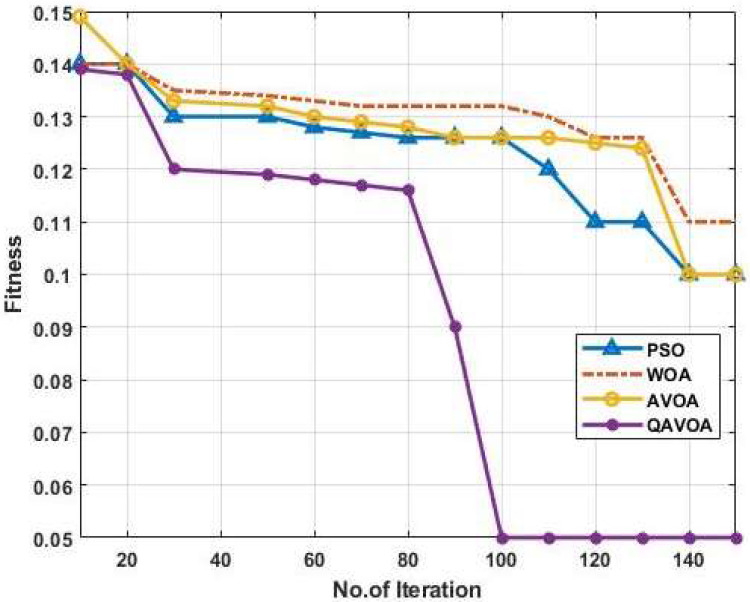
Fitness graph.

The performance of QAVOA-BPNN is analyzed using energy consumption, life expectancy, throughput, End to End Delay (EED), Packet Delivery Ratio (PDR) and Packet Loss Ratio (PLR). Here, the performances are analyzed with different optimization algorithms such as Particle Swarm Optimization, Whale Optimization Algorithm (WOA) and AVOA. The fitness graph for PSO, WOA, AVOA and QAVOA is shown in the Figure 4. The enhanced population diversity provided by the QOL in QAVOA is used to achieve a faster convergence than the PSO, WOA and AVOA.

#### 4.1.1. Energy Consumption

**Figure 5:**
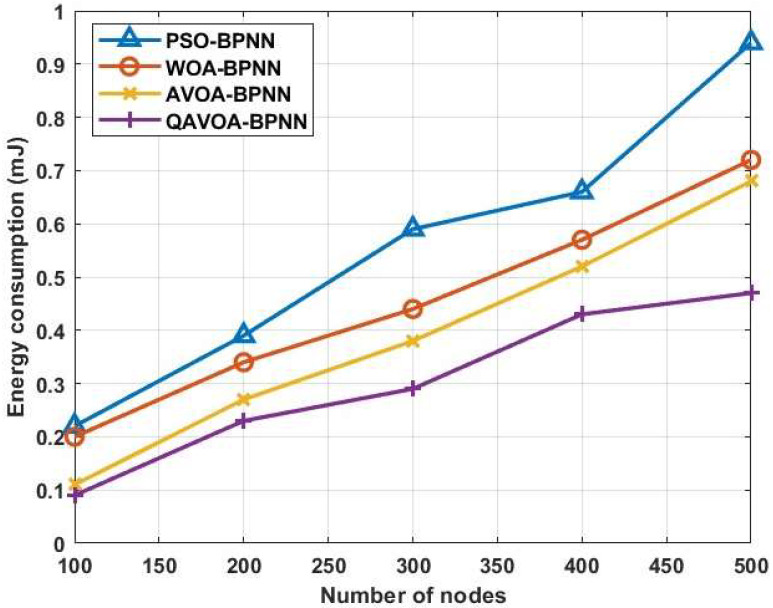
Energy consumption.

The amount of energy consumed by the nodes while transmitting and receiving the data packets is defined as energy consumption, which is formulated as Equation (33).

(33) $$Energy\;Consumption=E_{T}-\left(transmitted\;enrgy-received\;energy\right)$$

where, $E_{T}$ refers to the total amount of energy. Figure 5 and Table 2 show the comparison of energy consumption for QAVOA-BPNN with PSO-BPNN, WOA-BPNN and AVOA-BPNN. This analysis shows that the energy usage of QAVOA-BPNN is less when compared to the PSO-BPNN, WOA-BPNN and AVOA-BPNN. For example, the energy consumption for 500 nodes is 0.47 mJ, whereas the PSO-BPNN obtains 0.94 mJ, WOA-BPNN obtains 0.72 mJ and AVOA-BPNN 0.68 mJ. The following strategies are used to minimize the energy consumption: (1) data fusion using BPNN optimized using QAVOA is used to eliminate the redundant data and reduce the size of data, and (2) an optimal CH selection and route discovery using QAVOA.

#### 4.1.2. Life expectancy

**Figure 6:**
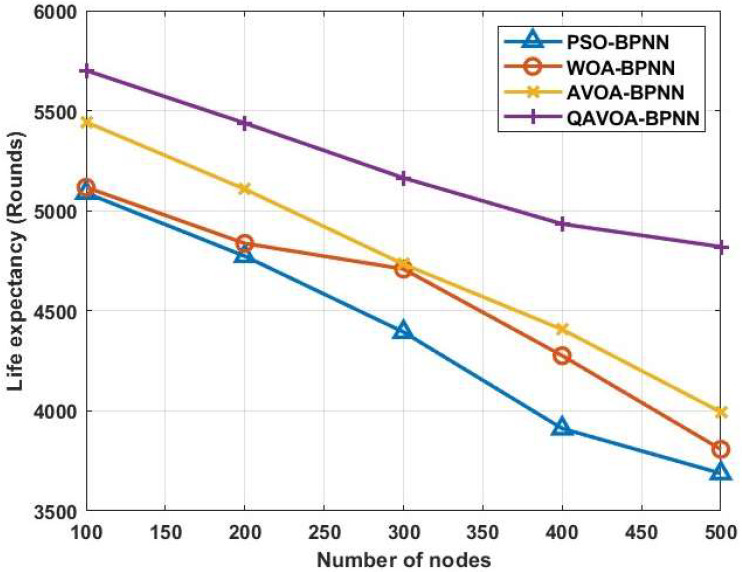
Life expectancy.

Life expectancy is the time at which all the sensors in the network lose their energy due to data transmitting over the WSN. The life expectancy comparison for QAVOA-BPNN with PSO-BPNN, WOA-BPNN and AVOA-BPNN is shown in Figure 6 and Table 3. The QAVOA-BPNN has higher life expectancy than the PSO-BPNN, WOA-BPNN and AVOA-BPNN. For example, the life expectancy for 500 nodes is 4820 rounds, whereas the PSO-BPNN obtains 3688 rounds, WOA-BPNN obtains 3808 rounds and AVOA-BPNN 3994 rounds. The lesser energy consumption achieved by the BPNN with QAVOA data fusion, and optimum CH and route discovery using QAVOA help to increase the life expectancy.

#### 4.1.3. Throughput

Throughput is defined as the number of packets that are successfully transmitted between the transmitter CHN to BS. The formula for the throughput is expressed in Equation (34).

(34) $$Throughput=\frac{Total\;number\;of\;packets\;delivered\;successfully}{Total\;time\;interval}$$

**Figure 7:**
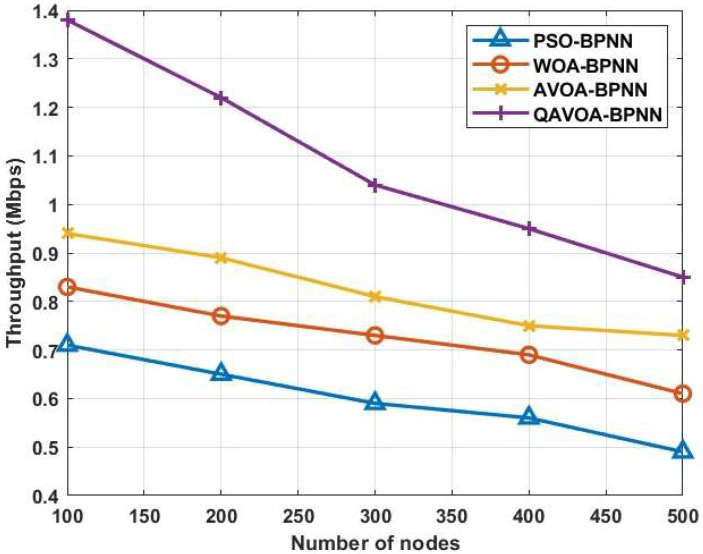
Throughput.

Figure 7 and Table 4 show the comparison of the throughput for QAVOA-BPNN with PSO-BPNN, WOA-BPNN and AVOA-BPNN. This analysis shows that the throughput of QAVOA-BPNN is high when compared to PSO-BPNN, WOA-BPNN and AVOA-BPNN. For example, the throughput for 500 nodes is 0.85 Mbps, whereas the PSO-BPNN obtains 0.49 Mbps, WOA-BPNN obtains 0.61 Mbps and AVOA-BPNN 0.73 Mbps. The enhanced population diversity achieved by QAVOA is used to perform an effective data fusion and reliable transmission over the WSN that helps to increase the amount of successful packets received by BS.

#### 4.1.4. End to End Delay

EED is defined as the amount of time taken to transmit the data from the transmitter CHN to BS, which is expressed as Equation (35).

(35) $$Delay=\frac{Sum\;of\;all\;packets\;delay}{Total\;number\;of\;received\;packets}$$

**Figure 8:**
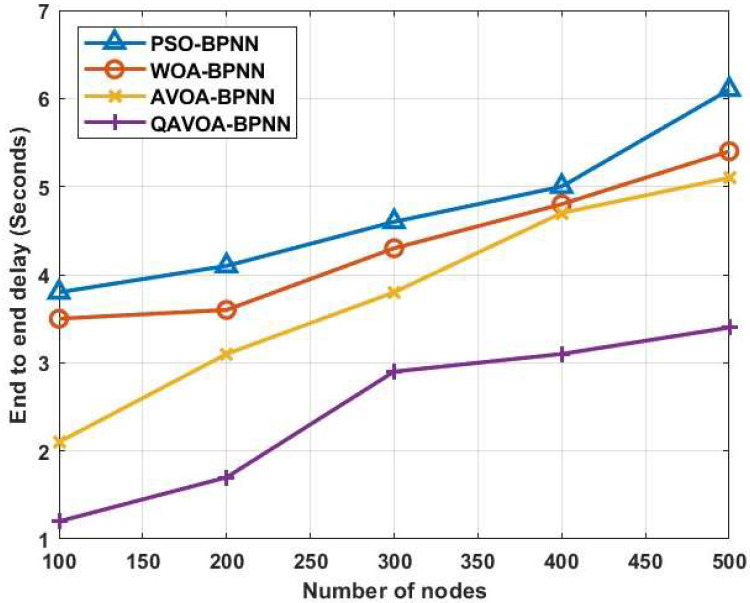
Delay.

The EED comparison for QAVOA-BPNN with PSO-BPNN, WOA-BPNN and AVOA-BPNN is shown in the Figure 8 and Table 5. The QAVOA-BPNN has lesser EED than the PSO-BPNN, WOA-BPNN and AVOA-BPNN. For example, the EED of 500 nodes is 3.4 s, whereas the PSO-BPNN obtains 6.1 s, WOA-BPNN obtains 5.4 s and AVOA-BPNN 5.1 s. The enhanced population diversity of QAVOVA helps to obtain the effective CH and route discovery along with data fusion using BPNN leads to transmitting the data packet with a lesser distance. The lesser transmission distance of QAVOA-BPNN helps to minimize the delay over the WSN.

#### 4.1.5. PDR and PLR

PDR is defined as the ratio among the amount of received packets and transmitted packets at Equation (36), whereas the PLR is the ratio among lost packets and transmitted packets, which is defined in Equation (37).

(36) $$PDR=\frac{Number\;of\;received\;packes}{Number\;of\;source\;packets}\times 100$$

(37) $$PLR=\frac{Number\;of\;lost\;packets}{Total\;number\;of\;sent\;packets}$$

**Figure 9:**
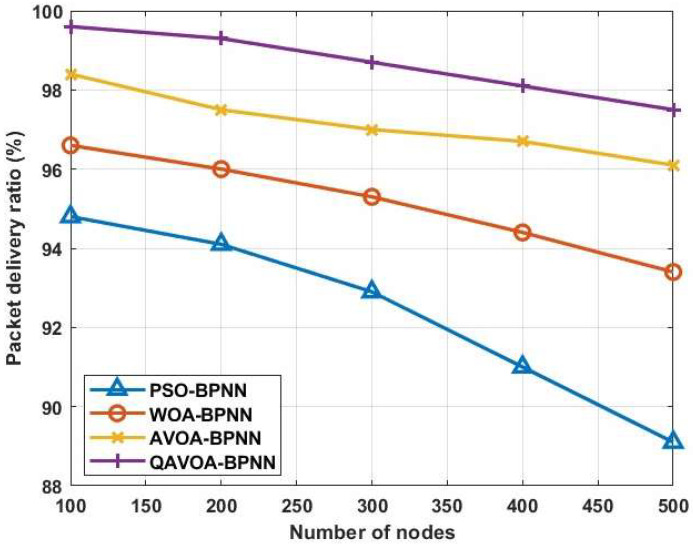
Packet delivery ratio.

**Figure 10:**
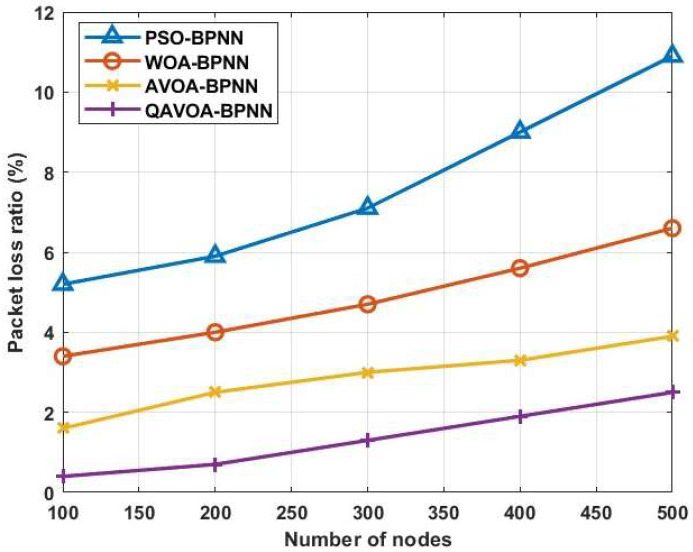
Packet loss ratio.

Figure 9 and Figure 10 shows the PDR and PLR analysis of QAVOA-BPNN, PSO-BPNN, WOA-BPNN and AVOA-BPNN. Further, Table 6 and Table 7 show the comparison of PDR and PLR, respectively. This analysis shows that the data delivery of the QAVOA-BPNN is improved when compared to the PSO-BPNN, WOA-BPNN and AVOA-BPNN. For example, the PDR of 500 nodes is 97.5%, whereas the PSO-BPNN obtains 89.1%, WOA-BPNN obtains 93.4% and AVOA-BPNN obtains 96.1%. The utilization of energy is decreased by fusing the data using BPNN optimized via QAVOA that helps to preserve the node’s energy. Therefore, the alive nodes of QAVOA-BPNN are increased, which helps to increase the data delivery over the network.

#### 4.1.6. Network Lifetime

**Figure 11:**
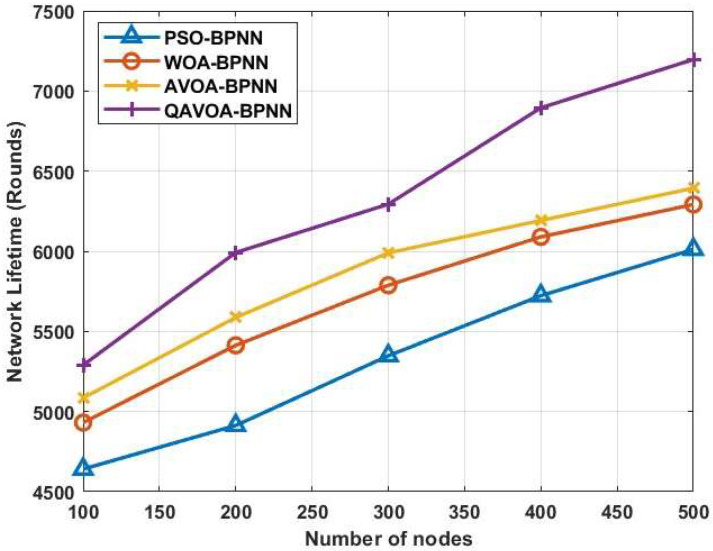
Network Lifetime.

In this approach, Network Lifetime predicts the amount of time between sensor node locations and a dead network. The network life spans of the QAVOA-BPNN that is being described and the current technology are contrasted in Figure 11. Data packets must travel farther and use more energy in a WSN. As the network time lengthens, data transmission efficiency becomes better. In comparison to the other methods like PSO-BPNN, WOA-BPNN, and AVOA-BPNN, which have life cycles of 6012, 6292, and 6394 rounds, respectively, the QAVOA-BPNN has a better network life cycle of 7197 (rounds) at 500 nodes. The comparison of Network Lifetime is shown in Table 8.

#### 4.1.7. Energy Efficiency

**Figure 12:**
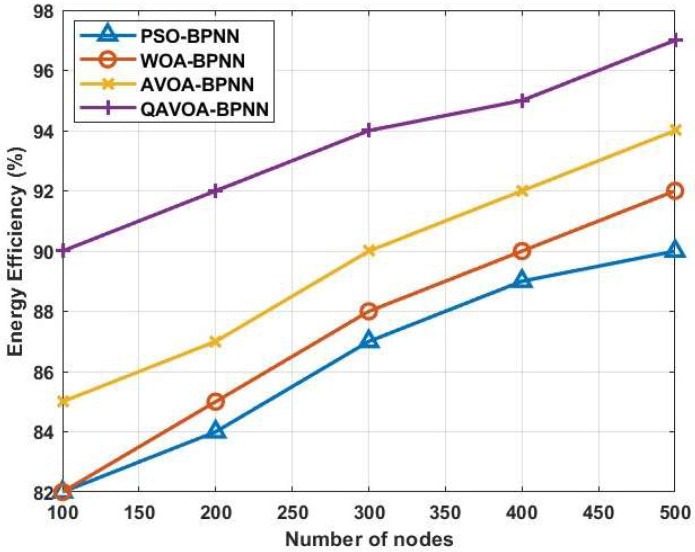
Energy efficiency.

The amount of energy that is still in use after network data transmission is complete is known as energy efficiency. Figure 12 shows the nodes energy efficiency for the PSO-BPNN, WOA-BPNN, AVOA-BPNN and QAVOA-BPNN methods. The simulation experiment is run using 500 nodes for this scenario. The QAVOA-BPNN technology performs with a 97.2% energy efficiency. The energy efficiency of other protocols like PSO-BPNN, WOA-BPNN, and AVOA-BPNN is 90.1%, 92.7%, and 94.4%, respectively. The comparison of energy efficiency is shown in Table 9. Analyses demonstrate that QAVOA-BPNN operates better than the others, as evidenced by its superior performance.

### 4.2. Comparative Analysis

The comparative analysis of the QAVOA-BPNN is provided in this section. The existing research such as CL-HHO [29] and ISSDE [30] are used to evaluate the QAVOA-BPNN. Table 10 shows the comparative analysis of QAVOA-BPNN with CL-HHO [29] and ISSDE [30]. From this comparison, it is determined that the QAVOA-BPNN achieves better performances than the CL-HHO [29] and ISSDE [30]. The PDR of the QAVOA-BPNN for 100 nodes is 99.6%, whereas the CL-HHO [29] obtains 99.4% and ISSDE [30] 98.2%. An effective data fusion obtained by the QAVOA-BPNN is used to eliminate the redundant data and minimized the size of the broadcasted data, which helps to minimize the energy consumption. Further, an optimum CH and route discovery using QAVOA is used to achieve reliable data transmission over the WSN.

### 4.3. Discussion

In order to eliminate redundant data and reduce the amount of broadcasted data in the WSN, optimized BPNN employing QAVOA-based data fusion is carried out in this article.

- Reliable data transmission over the WSN is achieved utilizing the shortest path generating method employing QAVOA.
- In order to reduce energy consumption and hence lengthen the expected lifespan, duplicated data are eliminated using QAVOA-BPNN, and the shortest paths are generated.
- The QAVOA-BPNN method is evaluated against existing techniques like Cross-Layer-based Harris Hawks Optimization (CL-HHO) and Improved Sparrow Search Utilizing Differential Evolution (ISSDE).
- The 500 nodes are thought to have been distributed at random over an area of 500 m×500 m.

- The QAVOA-BPNN is analyzed in terms of energy consumption (0.47 mJ), life expectancy (482 rounds), throughput (0.85 Mbps), End to End Delay (3.4 s), Packet Delivery Ratio (97.5%) and Packet Loss Ratio (2.5) Network Lifetime (7197 rounds) and Energy Efficiency (97.2%).

## 5. Conclusions

In this paper, optimized BPNN using QAVOA-based data fusion is performed for removing the redundant data and decreasing the amount of broadcasted data in the WSN. The BPNN performs effective data fusion by optimizing their weights and threshold coefficients, and the backward error propagation is used to minimize the error. Moreover, the optimum CHN from QAVOA is used to balance the energy consumption among the clusters. The shortest path generation using QAVOA is used to accomplish reliable data transmission over the WSN. Therefore, the redundant data elimination using QAVOA-BPNN, and the shortest path generation are used to minimize the energy utilization that leads to increase the life expectancy. From the results, it is determined that the QAVOA-BPNN achieves better performance than the CL-HHO and ISSDE. The life expectancy of QAVOA-BPNN for 500 nodes is 4820 rounds, which is high when compared to the CL-HHO and ISSDE.

In the future, a novel optimization algorithm can be used for improving the performances of WSN. Furthermore, the collision aware routing protocols will be used to avoid the congestion problem in the network. Moreover, the proposed QAVOA-based approach will be extended in the future to handle/analyze larger-scale WSNs with more complex data fusion and routing requirements.

## Figures and Tables

**Table 1:** Simulation parameters.

Parameter	Value
Number of nodes	500
Node distribution	Random
Network size	500 m×500 m
Location of BS	100, 100
Size of packet	4000 bits
Initial energy	0.1 J

**Table 2:** Comparison of energy consumption.

Methods	Energy Consumption (mJ)				
	100	200	300	400	500
**PSO-BPNN**	0.22	0.39	0.59	0.66	0.94
**WOA-BPNN**	0.20	0.34	0.44	0.57	0.72
**AVOA-BPNN**	0.11	0.27	0.38	0.52	0.68
**QAVOA-BPNN**	0.09	0.23	0.29	0.43	0.47

**Table 3:** Comparison of life expectancy.

Methods	Life Expectancy (Rounds)				
	100	200	300	400	500
**PSO-BPNN**	5089	4773	4394	3912	3688
**WOA-BPNN**	5117	4837	4709	4276	3808
**AVOA-BPNN**	5443	5108	4733	4407	3994
**QAVOA-BPNN**	5701	5438	5164	4934	4820

**Table 4:** Comparison of throughput.

Methods	Throughput (Mbps)				
	100	200	300	400	500
**PSO-BPNN**	0.71	0.65	0.59	0.56	0.49
**WOA-BPNN**	0.83	0.77	0.73	0.69	0.61
**AVOA-BPNN**	0.94	0.89	0.81	0.75	0.73
**QAVOA-BPNN**	1.38	1.22	1.04	0.95	0.85

**Table 5:** Comparison of EED.

Methods	End to End Delay (Seconds)				
	100	200	300	400	500
**PSO-BPNN**	3.8	4.1	4.6	5.0	6.1
**WOA-BPNN**	3.5	3.6	4.3	4.8	5.4
**AVOA-BPNN**	2.1	3.1	3.8	4.7	5.1
**QAVOA-BPNN**	1.2	1.7	2.9	3.1	3.4

**Table 6:** Comparison of PDR.

Methods	PDR (%)				
	100	200	300	400	500
**PSO-BPNN**	94.8	94.1	92.9	91.0	89.1
**WOA-BPNN**	96.6	96.0	95.3	94.4	93.4
**AVOA-BPNN**	98.4	97.5	97.0	96.7	96.1
**QAVOA-BPNN**	99.6	99.3	98.7	98.1	97.5

**Table 7:** Comparison of PLR.

Methods	PLR (%)				
	100	200	300	400	500
PSO-BPNN	5.2	5.9	7.1	9	10.9
WOA-BPNN	3.4	4	4.7	5.6	6.6
AVOA-BPNN	1.6	2.5	3	3.3	3.9
QAVOA-BPNN	0.4	0.7	1.3	1.9	2.5

**Table 8:** Comparison of Network Lifetime.

Methods	Network Lifetime (Rounds)				
	100	200	300	400	500
PSO-BPNN	4641	4913	5348	5723	6012
WOA-BPNN	4931	5413	5788	6090	6292
AVOA-BPNN	5085	5587	5990	6192	6394
QAVOA-BPNN	5290	5992	6294	6895	7197

**Table 9:** Comparison of energy efficiency.

Methods	Energy Efficiency (%)				
	100	200	300	400	500
PSO-BPNN	82.2	84.1	87.4	89.3	90.1
WOA-BPNN	82.3	85.4	88.6	90.2	92.7
AVOA-BPNN	85.6	87.2	90.4	92.5	94.4
QAVOA-BPNN	90.8	92.3	94.7	95.5	97.2

**Table 10:** Comparative analysis of QAVOA-BPNN.

Performances	Methods	Number of Nodes				
		100	200	300	400	500
**Energy consumption (mJ)**	**CL-HHO [29]**	0.1	0.25	0.33	0.47	0.55
	**ISSDE [30]**	0.12	0.30	0.36	0.51	0.62
	**QAVOA-BPNN**	0.09	0.23	0.29	0.43	0.47
**Life expectancy (Rounds)**	**CL-HHO [29]**	5600	5300	4900	4600	4100
	**ISSDE [30]**	5521	5207	4733	4366	3825
	**QAVOA-BPNN**	5701	5438	5164	4934	4820
**Throughput (Mbps)**	**CL-HHO [29]**	0.98	0.92	0.89	0.85	0.79
	**ISSDE [30]**	0.92	0.88	0.83	0.78	0.83
	**QAVOA-BPNN**	1.38	1.22	1.04	0.95	0.85
**EED (s)**	**CL-HHO [29]**	1.8	2.4	3.3	3.8	4.0
	**ISSDE [30]**	2.1	2.8	3.8	4.4	5.9
	**QAVOA-BPNN**	1.2	1.7	2.9	3.1	3.4
**PDR (%)**	**CL-HHO [29]**	99.4	98.4	97.8	96.5	95.5
	**ISSDE [30]**	98.2	97.6	97.0	95.9	93.1
	**QAVOA-BPNN**	99.6	99.3	98.7	98.1	97.5
**PLR (%)**	**CL-HHO [29]**	0.5	1	2	2.5	3.0
	**ISSDE [30]**	1.8	2.4	3	4.1	6.9
	**QAVOA-BPNN**	0.4	0.7	1.3	1.9	2.5

## Data Availability

The results of this study can be provided with reasonable request.
